# Alcohol use, suicidality and virologic non‐suppression among young adults with perinatally acquired HIV in Thailand: a cross‐sectional study

**DOI:** 10.1002/jia2.26064

**Published:** 2023-02-13

**Authors:** Linda Aurpibul, Pope Kosalaraksa, Surinda Kawichai, Pagakrong Lumbiganon, Pradthana Ounchanum, Wipaporn Natalie Songtaweesin, Tavitiya Sudjaritruk, Kulkanya Chokephaibulkit, Supattra Rungmaitree, Tulathip Suwanlerk, Jeremy L. Ross, Annette H. Sohn, Thanyawee Puthanakit, the Thai PAPAYA study team

**Affiliations:** ^1^ Research Institute for Health Sciences Chiang Mai University Chiang Mai Thailand; ^2^ Department of Pediatrics Faculty of Medicine Srinagarind Hospital Khon Kaen University Khon Kean Thailand; ^3^ Department of Pediatrics Faculty of Medicine Chulalongkorn University Bangkok Thailand; ^4^ Department of Pediatrics Chiangrai Prachanukroh Hospital Chiang Rai Thailand; ^5^ Department of Pediatrics and Clinical and Molecular Epidemiology of Emerging and Re‐emerging Infectious Diseases Research Cluster Faculty of Medicine Chiang Mai University Chiang Mai Thailand; ^6^ Department of Pediatrics Faculty of Medicine Siriraj Hospital Mahidol University Bangkok Thailand; ^7^ TREAT Asia/amfAR – The Foundation for AIDS Research Bangkok Thailand

**Keywords:** alcohol, suicidality, virologic non‐suppression, young adults, perinatal HIV infection, Asia

## Abstract

**Introduction:**

Young adults with perinatally acquired HIV (YA‐PHIV) are facing transitions to adult life. This study assessed health risk behaviours (including substance use), mental health, quality of life (QOL) and HIV treatment outcomes of Thai YA‐PHIV.

**Methods:**

A cross‐sectional study was conducted in Thai YA‐PHIV aged 18–25 years who were enrolled in a prospective cohort study at five tertiary paediatric HIV care centres in Thailand. Study data were obtained through face‐to‐face interviews from November 2020 to July 2021. Assessments were performed for alcohol use (Alcohol Use Disorders Identification Test; AUDIT), smoking (Fagerstrom Test for Nicotine Dependence), drug/substance use (Drug Abuse Screening Test; DAST‐10), depression (Patient Health Questionnaire for Adolescents; PHQ‐A), anxiety (Generalized Anxiety Disorder; GAD‐7) and QOL (World Health Organization QOL Brief‐Thai). HIV treatment outcomes were extracted from the National AIDS Program database.

**Results:**

Of 355 YA‐PHIV, 163 (46%) were males: their median age was 21.7 (interquartile range, IQR 20.2–23.5) years. There were 203 YA‐PHIV (58%) who reported ever having sex; 141 (40%) were sexually active in the past 6 months, of whom 86 (61%) reported 100% condom use. Overall, 49 (14%) met the criteria for harmful alcohol use; 28 (7.9%) were alcohol dependent. Sixty (17%) were current smokers and 37 (11%) used drugs/substances. The frequency of moderate up to severe symptoms for depression was 18% and for anxiety was 9.7%. Their overall QOL was good in 180 (51%), moderate in 168 (47%) and poor in five (1.4%). There were 49 YA‐PHIV (14%) with CD4 <200 cells/mm^3^ and 85 (24%) with virologic non‐suppression (HIV‐RNA >200 copies/ml). On multivariate analyses, the highest education at the primary to high school or vocational school levels (adjusted odds ratio [aOR] 2.02, 95% CI 1.40–3.95, *p* 0.04), harmful alcohol use (aOR 2.48, 95% CI 1.24–4.99, *p* 0.01), alcohol dependence (aOR 3.54, 95% CI 1.51–8.31, *p* <0.01) and lifetime suicidal attempt (aOR 2.66, 95% CI 1.11–6.35, *p* 0.03) were associated with non‐suppression.

**Conclusions:**

Regular screening for alcohol use and mental health, including suicidality, would be useful to identify YA‐PHIV who need more intensive psychosocial support or referral services to ensure they can achieve and maintain a high QOL into adult life.

## 1 INTRODUCTION

After almost two decades of national scale‐up of antiretroviral treatment in Thailand, children with perinatally acquired HIV have grown up into young adults (YA‐PHIV). Health risk behaviours increasingly reported among YA‐PHIV include the use of alcohol, other substances and tobacco [1, 2], which have been associated with reduced adherence and unfavourable treatment outcomes [3]. Mental health problems are also increasing, which can negatively impact antiretroviral treatment (ART) adherence [4, 5, 6, 7, 8]. Studies of quality of life (QOL) in YA‐PHIV have had mixed results, with some reporting lower [9], unchanged [10] and better QOL [11] compared to HIV‐negative youth, depending on the domains assessed.

YA‐PHIV additionally have had to face multiple challenges that extended beyond maintaining their physical health, including HIV‐related stigma [12] and familial and economic insecurities [13]. The combination of poorer health and social instability has led some YA‐PHIV to fall behind in their formal education [14, 15], which could negatively impact their ability to function as independent adults in society.

Data on the health risk behaviours, mental health and QOL of Asian YA‐PHIV and their association with treatment outcomes would provide valuable insights into how HIV programmes can better target their resources to provide more effective support to YA‐PHIV as they age. While most Thai YA‐PHIV were transitioned out of paediatric clinics to either adult clinics in the same hospitals or other HIV care facilities around the age of 15 or older following the decentralization policy [16], Thailand's relatively longer history of ART scale‐up in the region presents an opportunity to study these outcomes in an older cohort.

## 2 METHODS

### 2.1 Study design and recruitment

We conducted a cross‐sectional study at five tertiary paediatric HIV care centres in Bangkok (two sites), Chiang Mai, Chiang Rai and Khon Kaen, Thailand. Inclusion criteria were: (1) aged 18–25 years at enrolment; (2) had perinatally acquired HIV infection; and (3) initiated ART at the paediatric HIV clinic at the study sites before transitioning to an adult clinic, or remained in the paediatric clinics. From November 2020 to July 2021, 811 potential participants who were previously under care at the clinics were identified from existing databases at each participating site. The study team contacted 586 (72%) by approaching them during regular follow‐up care or by making phone calls to those already transferred to other clinics. Due largely to COVID‐19 travel restrictions, 355 (61%) YA‐PHIV were able to come to a clinic in person to participate in the study. Those with cognitive disabilities or who had active acute illnesses were excluded. The study was approved by the institutional review boards of each study site. Written informed consent was obtained before enrolment.

### 2.2 Data collection and tools

Baseline data at cohort enrolment were used in this analysis. Participant data on the most recent CD4 count and HIV RNA within the past 12 months after enrolment were collected from the National AIDS Program database. Viral non‐suppression was defined as having an HIV RNA >200 copies/ml [17]. Data on socio‐demographic information and sexual behaviours were collected from participating YA‐PHIV by study staff during face‐to‐face interviews (total 15–20 minutes). They were asked to self‐complete six paper‐based questionnaires about their health risk behaviours, mental health and QOL.

1) The Alcohol Use Disorders Identification Test (AUDIT) was used to assess alcohol use. Harmful alcohol use was defined as having AUDIT scores ≥8 in both sexes, and alcohol dependence was defined as having AUDIT scores ≥13 in females and ≥15 in males [18, 19].
2) The Drug Abuse Screening Test (DAST‐10) was used to assess substance use, excluding alcohol and smoking, in the past 12 months. The degree of problems related to drug abuse was classified by score as low (1–2), moderate (3–5) and substantial (≥6) [20].
3) The Fagerstrom Test for Nicotine Dependence was used to assess smoking, Nicotine dependence was defined by score as low (1–4), moderate (5–7) and high (≥8) [21].
4) The Patient Health Questionnaire for Adolescents (PHQ‐A, validated Thai version) was used to assess depressive symptoms in the past 2 weeks, with questions on suicidality in the past month and over a lifetime. Depressive symptoms were classified by score as mild (5–9), moderate (10–14), moderately severe (15–19) and severe (20–27). A PHQ‐A score ≥10 was considered to represent significant depression [22, 23].
5) The Generalized Anxiety Disorder 7‐item test (GAD‐7, validated Thai version) was used to assess anxiety symptoms, classified by scores as minimal (1–4), mild (5–9), moderate (10–14) and severe (15–21). Scores ≥10 were considered significant [24].
6) The World Health Organization QOL instrument (WHO QOL‐BREF, validated Thai version) was used to assess their QOL over the past 2 weeks across four domains: physical health, psychological health, social relationships and environment [25]. QOL was categorized by score as poor (26–60), moderate (61–95) and good (96–130) [26].

### 2.3 Statistical analysis

Categorical variables are presented as counts and percentages, and continuous variables as medians with interquartile ranges (IQR). Pearson's chi‐square test, Fisher's exact test and Z‐test were utilized to assess for associations between variables. Factors associated with virologic non‐suppression were assessed using univariate and multivariate logistic regression, and presented as odds ratios with a 95% confidence interval (CI) and Z‐test *p*‐values. Variables with *p* <0.1 in the univariate analysis were included in the multivariate analysis. Stata/SE version 13.0 was used for all data analyses.

## 3 RESULTS

Three hundred and fifty‐five YA‐PHIV were enrolled (Table 1); 163 (4%) were males. Their median (IQR) age was 21.7 (20.2–23.5) years. At enrolment, 198 (56%) were still being followed at their paediatric clinic, 49 (14%) had moved to a public sector adult HIV clinic, 100 (28%) had been transferred to other hospitals under the national HIV treatment programme, two (0.6%) went to private HIV clinics and six (1.7%) were not in active HIV care. Overall, 253 (71%) completed between primary to high school or vocational school as their highest level of formal education, while 102 (29%) attended university; 163 (46%) had a full‐time job; 320 (90%) were single; and 197 (56%) lived with parents/relatives. Among the 203 YA‐PHIV (58%) who reported ever having sex, the median (IQR) age of sexual debut was 18.0 (16.0–19.0) years. Their self‐reported sexual orientation was heterosexual in 171 (86%), homosexual in 17 (8.5%) and bisexual in 11 (5.5%). Of the 141 (40%) who were sexually active in the past 6 months, 86 (61%) reported 100% condom use. There were 207 (58%) who reported ever drinking alcohol, with 49 (14%) who drank at a harmful level, while 13 (6.8%) females and 15 (9.2%) males were alcohol dependent (scores ≥15 and 13, respectively). There were 37 (11%) YA‐PHIV who reported using drugs/substances other than alcohol and tobacco. Sixty (17%) were current smokers, of whom 45 (13%) had low and 15 (4.2%) had moderate nicotine dependency.

**Table 1:** Demographic characteristics, health risk behaviours and life experiences of Thai young adults with perinatally acquired HIV, stratified by sex

Characteristics	Total	Female	Male
Number of participants	355	192 (54)	163 (46)
Demographic characteristics			
Age, median (IQR)	21.7 (20.2, 23.5)	21.8 (20.1, 23.5)	21.7 (20.2, 23.5)
18–19.9 years	83 (23)	47 (25)	36 (22)
20–25.5 years	272 (77)	145 (76)	127 (78)
Highest education at the time of interview			
Primary to high school or vocational school	253 (71)	126 (56)	127 (78)
University/bachelor/or higher	102 (29)	66 (34)	36 (22)
Employment status			
Full‐time	163 (46)	82 (43)	81 (50)
Part‐time/occasionally	49 (14)	25 (13)	24 (15)
Seeking job/not working	41 (12)	27 (14)	14 (8.6)
Studying (not working)	102 (29)	58 (30)	44 (27)
Marital status			
Single/no partner	212 (60)	102 (53)	110 (68)
Single, had partner(s)	108 (30)	66 (34)	42 (26)
Married and living together	28 (7.9)	19 (9.9)	9 (5.5)
Divorced/separated/others	7 (2.0)	5 (2.6)	2 (1.2)
Housing status			
No expense (Family house)	197 (56)	101 (53)	96 (59)
Renting (Dorm/apartment)	107 (30)	64 (33)	43 (26)
Others	51 (14)	27 (14)	24 (15)
Sexual behaviours			
Ever had sex (*N* = 353)			
Yes	203 (58)	109 (57)	94 (58)
No	150 (43)	82 (43)	68 (42)
Age at sexual debut of those who ever‐had sex (*N* = 200)			
≤16 years	66 (33)	29 (27)	37 (40)
17–19 years	89 (45)	50 (47)	39 (42)
≥20 years	45 (23)	28 (26)	17 (18)
Self‐reported sexual orientation (*N* = 199)			
Heterosexual	171 (86)	91 (87)	80 (85)
Homosexual	17 (8.5)	5 (4.8)	12 (13)
Bisexual	11 (5.5)	9 (8.6)	2 (2.1)
Condom use among those who were sexually active in the past 6 months (*N* = 141)			
100%	86 (61)	48 (61)	38 (61)
Not 100%	55 (39)	31 (39)	24 (39)
Ever been or known to have made someone pregnant	*N* = 201		
Yes	50 (25)	32 (29)	18 (20)
No	151 (75)	77 (71)	74 (80)
Alcohol, drug/substance uses and smoking			
Alcohol drinking habit (AUDIT)			
No drinking	148 (42)	94 (49)	54 (33)
Drinking but not harmful (score <8)	130 (37)	69 (36)	61 (37)
Drinking at a harmful level (score ≥8)	49 (14)	16 (8.3)	33 (20)
Alcohol dependence (score ≥13 in females, ≥15 in males)	28 (7.9)	13 (6.8)	15 (9.2)
Drug/substance use (DAST‐10)			
No use	316 (90)	172 (91)	144 (88)
Low (scores 1–2)	26 (7.4)	18 (9.5)	8 (4.9)
Moderate (scores 3–5)	9 (2.6)	–	9 (5.5)
Substantial (scores ≥6)	2 (0.6)	–	2 (1.2)
Smoking behaviour (Fagerstrom)			
No smoking	295 (83)	186 (97)	109 (67)
Current smokers	60 (17)	6 (3.1)	54 (33)
Low nicotine dependence (scores 1–4)	45 (13)	5 (2.6)	40 (25)
Moderate nicotine dependence (scores 5–7)	15 (4.2)	1 (0.5)	14 (8.6)

Abbreviations: AUDIT, Alcohol Use Disorder Identification Test; DAST‐10, Drug Abuse Screening Test; Fagerstrom, Test for Nicotine Dependence; IQR, interquartile range.

*p*‐Value for chi‐square or Fisher's exact test.

Overall, 63 (18%) had depressive symptoms (PHQ‐A ≥ 10): 46 (13%) moderate and 17 (4.8%) severe; 34 (9.6%) had suicidal ideation within the past month, and lifetime suicidal attempts were reported by 28 (7.9%) (Table 2). Anxiety symptoms (GAD‐7 ≥ 10) were found in 25 (9.7%): 19 (7.4%) moderate and six (2.3%) severe. Overall QOL among YA‐PHIV was good in 180 (51%), moderate in 168 (47%) and poor in five (1.4%). The domains with the highest scores were psychological (good in 56%), followed by physical (good in 45%) and environmental (good in 39%). There were 49 (14%) YA‐PHIV with CD4 counts <200 cells/mm^3^ and 85 (24%) with virologic non‐suppression.

**Table 2:** Mental health, quality of life and HIV treatment outcomes of young adults with perinatally acquired HIV, stratified by sex

Variables	Total	Female	Male
Number of participants	355	192	163
Mental health			
Depressive symptoms (PHQ‐A)			
Mild (5–9)	133 (38)	69 (36)	64 (40)
Moderate (10–14)	46 (13)	22 (12)	24 (15)
Moderately severe (15–19)	11 (3.1)	5 (2.6)	6 (3.7)
Severe (20–27)	6 (1.7)	5 (2.6)	1 (0.6)
Suicidality			
Had suicide ideation in the last month			
Yes	34 (9.6)	22 (12)	12 (7.4)
No	319 (90)	169 (89)	150 (93)
Lifetime attempted suicide			
Yes	28 (7.9)	17 (8.9)	11 (6.8)
No	325 (92)	174 (91)	151 (93)
Anxiety symptoms (GAD‐7)			
Minimal (scores 1–4)	163 (64)	91 (63)	72 (64)
Mild (scores 5–9)	68 (27)	36 (25)	32 (29)
Moderate (scores 10–14)	19 (7.4)	14 (9.7)	5 (4.5)
Severe (scores 15–21)	6 (2.3)	3 (2.1)	2 (2.7)
Quality of life (WHO‐QOL BREF)			
Physical			
Good (27–35)	158 (45)	83 (44)	75 (46)
Moderate (17–26)	195 (54)	108 (55)	87 (54)
Poor (7–16)	2 (0.6)	0	2 (1.2)
Psychological			
Good (23–30)	199 (56)	112 (59)	87 (54)
Moderate (15–22)	145 (41)	79 (38)	75 (43)
Poor (6–14)	9 (2.6)	5 (2.6)	4 (2.5)
Social			
Good (12–15)	142 (40)	73 (38)	69 (43)
Moderate (8–11)	211 (53)	118 (54)	93 (51)
Poor (3–7)	25 (7.1)	16 (8.3)	9 (5.5)
Environment			
Good (30–40)	138 (39)	74 (39)	64 (40)
Moderate (19–29)	215 (58)	117 (58)	98 (59)
Poor (8–18)	9 (2.6)	6 (3.1)	3 (1.8)
Overall QOL			
Good (96–130)	180 (51)	99 (52)	81 (50)
Moderate (61–95)	173 (48)	92 (47)	81 (48)
Poor (26–60)	5 (1.4)	2 (1.0)	3 (1.8)
HIV treatment outcomes			
CD4 count (cells/mm^3^)			
≥351	266 (76)	151 (80)	115 (72)
201–350	35 (10)	16 (8.4)	19 (12)
≤200	49 (14)	23 (12)	26 (16)
Plasma HIV RNA >200 copies/ml	85 (24)	41 (22)	44 (27)

Abbreviations: GAD‐7, Generalized Anxiety Disorder 7‐Items; IQR, interquartile range; PHQ‐A, the Patient Health Questionnaire for Adolescents; WHO‐QOL‐BREF, the World Health Organization Quality of Life, Field Trial Version.

*p*‐Value for chi‐square or Fisher's exact test.

On multivariate logistic regression, virologic non‐suppression was associated with primary to high school or vocational school education (adjusted odds ratio [aOR] 2.02, 95% CI 1. 40–3.95, *p* 0.04), harmful alcohol use (aOR 2.48, 95% CI 1.24–4.99, *p* 0.01), alcohol dependence (aOR 3.54, 95% CI 1.51–8.31, *p* <0.01) and lifetime suicidal attempt (aOR 2.66, 95% CI 1.11–6.35, *p* = 0.03) (Table 3).

**Table 3:** Logistic regression analysis on virologic non‐suppression (HIV RNA level >200 copies/ml)

			Bivariate analysis		Multivariate analysis	
Variables	*N*	Virologic non‐suppression, *n* (%)	Crude OR (95% CI)	*p*‐value**^b^**	Adjusted OR (95% CI)	*p*‐value**^b^**
Number	353^a^	85 (24)				
Age (years)		As cont. var	0.94 (0.83, 1.06)	0.32		
Sex						
Female	191	41 (22)	1			
Male	162	44 (27)	1.36 (0.84, 2.22)	0.21		
Marital status						
Not married	318	72 (23)	1			
Married	35	13 (37)	2.02 (0.97, 4.21)	0.06	1.77 (0.78, 3.97)	0.17
Highest education						
University or higher	102	14 (14)	1			
Primary to high school or vocational school	251	71 (28)	2.48 (1.32, 4.64)	0.01	2.02 (1.04, 3.95)	0.04
Employment status						
Studying/others	119	19 (16)	1			
Seeking (no job)	23	8 (35)	2.81 (1.04, 7.54)	0.04		
Have job (full/part‐time/occasional)	211	58 (28)	2.00 (1.12, 3.55)	0.02		
Alcohol use (AUDIT)						
No/yes and not harmful	276	52 (19)	1			
Yes, and harmful	49	19 (39)	2.73 (1.42, 5.22)	<0.01	2.48 (1.24, 4.99)	0.01
Yes, and dependent	28	14 (50)	4.31 (1.94, 9.58)	<0.01	3.54 (1.51, 8.31)	<0.01
Smoking behaviours (Fagerstrom)						
Not current smoker	294	64 (22)	1			
Low dependence (0–4)	44	15 (34)	1.86 (0.94, 3.68)	0.08		
Moderate dependence (5–7)	15	6 (40)	2.40 (0.82, 6.98)	0.11		
Drug/substance use (DAST‐10)						
No	314	76 (24)	1			
Yes	39	9 (23)	0.94 (0.43, 2.07)	0.88		
Ever had sex						
No	148	29 (20)	1			
Yes	203	56 (28)	1.56 (0.94, 2.60)	0.09		
Had sex in the past 6 months						
No	210	44 (21)	1			
Yes	141	41 (29)	1.55 (0.94, 2.53)	0.08		
Condom use among those who had sex in the past 6 months						
100%	86	21 (24)	1			
Not 100%	55	20 (36)	1.77 (0.85, 3.70)	0.13		
Depression symptoms (PHQ‐A)						
No depression (0–4)	157	24 (15)				
Mild (5–9)	131	44 (34)	2.80 (1.59, 4.94)	0.000		
Moderate/severe (10–27)	63	17 (27)	2.05 (1.01, 4.15)	0.047		
Mild/moderate/severe (≥5)	194	61 (31)	2.54 (1.50, 4.32)	<0.01	1.71 (0.93, 3.16)	0.08
Suicidal ideation in the past month						
No	318	74 (23)	1			
Yes	33	11 (33)	1.65 (0.76, 3.56)	0.20		
Lifetime suicidal attempt						
No	323	72 (22)	1			
Yes	28	13 (46)	3.02 (1.37, 6.64)	<0.01	2.66 (1.11, 6.35)	0.03
Anxiety symptoms (GAD‐7)						
Minimal to mild (<10)	325	77 (24)	1			
Moderate to severe (≥10)	25	7 (28)	1.25 (0.50, 3.11)	0.63		
Quality of life, WHO‐QOL‐BREF	351^c^					
Physical health						
Good	157	28 (18)				
Moderate/poor	194	57 (29)	1.92 (1.15, 3.20)	0.01		
Psychological						
Good	198	40 (20)				
Moderate/poor	153	45 (29)	1.64 (1.01, 2.69)	0.47		
Social relationships						
Good	142	29 (20)				
Moderate/poor	209	56 (27)	1.43 (0.86, 2.38)	0.17		
Environment						
Good	138	27 (20)	1			
Moderate/poor	213	58 (27)	1.54 (0.92, 2.58)	0.10		
Overall quality of life						
Good (96–125)	179	32 (18)	1			
Moderate (62–95)	167	50 (30)	1.96 (1.18, 3.26)	0.01	1.22 (0.69, 2.18)	0.49
Poor (41–60)	5	3 (60)	6.89 (1.10, 42.94)	0.04	4.22 (0.60, 29.48)	0.15

Abbreviations: AUDIT, the Alcohol Use Disorder Identification Test; DAST‐10, the Drug Abuse Screening Test; Fagerstrom, the Test for Nicotine Dependence; GAD‐7, Generalized Anxiety Disorder 7‐Items; PHQ‐A, the Patient Health Questionnaire for Adolescents; WHO‐QOL‐BREF, the World Health Organization Quality of Life, Validated Thai version.

^a^ Two participants had missing HIV RNA.

^b^ All variables with *p* <0.10 from the univariate model were included in the multivariate analysis, employment status was excluded due to collinearity with education.

^c^ Two participants with missing HIV RNA and two who did not complete WHO‐QOL‐BREF were excluded.

## 4 DISCUSSION

Alcohol use and suicidality were associated with poorer treatment outcomes among Thai YA‐PHIV. More than half of study participants used alcohol, reflecting increasing use with age when compared to data on younger adolescents with HIV in the region. A 2014 study in Thailand, Malaysia and Vietnam reported that 32% of adolescents at the median age of 14.5 years drank alcohol [27]. The frequency and severity of alcohol use among Thai YA‐PHIV in this study was higher than in a Kenyan study in adolescents and young adults with HIV who were at a similar median age of 21 years [28]. They reported alcohol use among 13%, with 5.4% drinking at harmful and 2.0% at dependent levels. Alcohol use also is subject to other aspects of the local context. While the Thai National survey in 2013 reported prevalence of alcohol use in those 13–24 years was 28% [29], a community‐based survey in rural Thailand reported alcohol use among adolescents aged 12–18 years as high as 47% [30]. Nevertheless, the association of harmful or dependent alcohol use with virologic non‐suppression in our cohort emphasizes the importance of early intervention among YA‐PHIV. The frequency of smokers (17%) in our study was slightly lower than that in general Thai youth aged between 13 and 24 years (23%) [29]. A reduction in the number of smokers has been observed nationwide following years of anti‐smoking campaigns in Thailand, while the number of new alcohol drinkers still increased [31, 32].

Reports of depression and anxiety among adolescents and YA‐PHIV vary by age, geographic context and screening tool used. The 18% and 9.7% prevalences of depressive and anxiety symptoms, respectively, in our study were similar to previous Thai studies that reported 15% depression and 10% anxiety in Bangkok [33]; and 11% depression and 7% anxiety in Chiang Mai [34]. Both studies assessed youth with PHIV at the median age of 19 years. Another study in Northeast Thailand reported depressive disorders in 18% of YA‐PHIV (median age 17.5 years) and documented an association with virologic failure [35]. A Chinese study in adolescents with HIV (mean age 16 years) reported a prevalence of depressive symptoms at 32% [36]. Moreover, depressive symptoms were reported among 61% of Kenyan adolescents and young adults with HIV (median age 21 years) [37].

In terms of suicidality, a school‐based student health survey in Thailand reported the overall prevalence of suicidal ideation at 8.8% in 2008 [38], which increased to 20.5% in 2015—twice the rate found in our study [39]. Nevertheless, having a history of prior suicide attempt was associated with virologic non‐suppression, emphasizing the importance of including suicidality assessments in the context of comprehensive mental health screening for YA‐PHIV to identify those at risk.

The majority of YA‐PHIV reported either good or moderate overall QOL. Similar to mental health screening data, studies of the association between QOL and HIV outcomes differed by context and population. A Spanish study in youth with PHIV reported worse QOL using the SF‐12 tool across domains of physical and mental health in comparison to HIV‐negative youth [40]. In contrast, a Dutch study using the PedSqL tool reported better QOL in youth with PHIV compared to HIV‐negative youth in most domains tested [11]. Longitudinal assessment of QOL using the same tool in the same population may be needed for more accurate assessments over time. The virologic suppression rate (76%) in our study compares to reports from the UK (84%) and the United States (60%) [41, 42]. The UNAIDS third 90 target remains difficult for YA‐PHIV to reach, which supports the need for more attention to this population [43]. While only 61% of sexually active participants used condoms 100% of the time, increased promotion of condom use could minimize the risk of HIV and other sexual‐transmitted infection transmission, especially in those who are not virally suppressed.

A strength of this study was that it was conducted at multiple sites and contexts in Thailand, increasing our ability to apply our findings more broadly to YA‐PHIV nationwide and compare them to other countries in the region. However, limitations included lacking age‐matched control groups who were exposed or unexposed to HIV, and socio‐demographic and economic data from families during childhood or early adolescence. This prevents us from assessing whether shared environments differentially impacted siblings growing up without HIV. In addition, there was a risk of selection bias, as participants were those who had been retained in care at study sites prior to enrolment, and may consequently have had better health outcomes or less frequent risk behaviours than YA‐PHIV who were not in care at all or who returned to care later as adults. There was also a risk of social desirability bias in the self‐reported screening results due to the sensitive nature of the topics raised. The study was conducted during the COVID‐19 pandemic, which may have affected symptoms and perspectives on mental health and QOL among the YA‐PHIV. Due to the cross‐sectional design, we could not draw causal inferences between health risk behaviours, mental health and treatment outcomes. Nevertheless, it remains notable that over half of Thai YA‐PHIV in our cohort used alcohol, and significant associations between alcohol use, suicidality and virologic non‐suppression were observed.

## 5 CONCLUSIONS

Mental health and health risk behaviour screening should be incorporated into routine care for YA‐PHIV to identify those who need additional psychiatric assessment and prevention interventions in order for them to maintain HIV disease control and thrive in their communities. Helping them to achieve and sustain the highest possible QOL and other health outcomes should be some of the ultimate goals for HIV care of YA‐PHIV.

## COMPETING INTERESTS

The authors have no competing interests.

## AUTHORS’ CONTRIBUTIONS

LA, PK, PL, PO, TP and KC: Study design, study conducting, data collection, data cleaning, data interpretation, manuscript preparation and reviewing.

SK: Data analysis plan, statistical analysis, manuscript preparation and reviewing.

WNS, TS and SR: Study conducting, data collection, data interpretation and manuscript reviewing.

TS, JLR and AHS: Study design, data collecting plan, sponsor coordination, data interpretation and manuscript reviewing.

## FUNDING

This study is supported by grants to amfAR from ViiV Healthcare, and U.S. National Institutes of Health's National Institute of Allergy and Infectious Diseases, the *Eunice Kennedy* Shriver National Institute of Child Health and Human Development, the National Cancer Institute, the National Institute of Mental Health, the National Institute on Drug Abuse, the National Heart, Lung, and Blood Institute, the National Institute on Alcohol Abuse and Alcoholism, the National Institute of Diabetes and Digestive and Kidney Diseases, and the Fogarty International Center, as part of the International Epidemiology Databases to Evaluate AIDS (IeDEA; U01AI069907).

## DISCLAIMER

The content is solely the responsibility of the authors and does not necessarily represent the official views of amfAR, ViiV Healthcare or the U.S. National Institutes of Health.

## Data Availability

The data that support the findings of this study are available from the corresponding author upon reasonable request.
